# How Random Is Social Behaviour? Disentangling Social Complexity through the Study of a Wild House Mouse Population

**DOI:** 10.1371/journal.pcbi.1002786

**Published:** 2012-11-29

**Authors:** Nicolas Perony, Claudio J. Tessone, Barbara König, Frank Schweitzer

**Affiliations:** 1 Chair of Systems Design, ETH Zurich, Zurich, Switzerland; 2 Department of Animal Behaviour, Institute of Evolutionary Biology and Environmental Studies, University of Zurich, Zurich, Switzerland; Pennsylvania State University, United States of America

## Abstract

Out of all the complex phenomena displayed in the behaviour of animal groups, many are thought to be emergent properties of rather simple decisions at the individual level. Some of these phenomena may also be explained by random processes only. Here we investigate to what extent the interaction dynamics of a population of wild house mice (*Mus domesticus*) in their natural environment can be explained by a simple stochastic model. We first introduce the notion of perceptual landscape, a novel tool used here to describe the utilisation of space by the mouse colony based on the sampling of individuals in discrete locations. We then implement the behavioural assumptions of the perceptual landscape in a multi-agent simulation to verify their accuracy in the reproduction of observed social patterns. We find that many high-level features – with the exception of territoriality – of our behavioural dataset can be accounted for at the population level through the use of this simplified representation. Our findings underline the potential importance of random factors in the apparent complexity of the mice's social structure. These results resonate in the general context of adaptive behaviour versus elementary environmental interactions.

## Author Summary

From the synchronised beauty of fish schools to the rigorous hierarchy of ant colonies, animals often display awe-inspiring collective behaviour. In recent years, principles of statistical physics have helped to unveil some simple mechanisms behind the emergence of such collective dynamics. Among the most elementary tools used to explain group behaviour are random processes, a typical example being the so-called “random walk”. In this paper, we have developed a framework based on such random assumptions to study the spatial and social structure of a population of wild house mice. We introduce the concept of perceptual landscape to describe the spatial behaviour of animals, whilst including all sensory and social constraints they are subject to: the perceptual landscape effectively maps the environment of animals as they perceive it. By applying our assumptions to a multi-agent model, we are able to reveal that much of the high-level social behaviour observed in the mouse population can indeed be explained through the many interactions of randomly moving individuals. This raises the question of how much of what we often regard as complex natural phenomena may, in fact, be the result of exceedingly simple forces.

## Introduction

In a famous passage of his book *The Sciences of the Artificial* [1] the sociologist Herbert Simon considers the winding, weaving path of an ant as it makes its journey home across the rugged landscape of a wind- and wave-beaten sandy beach. He notes that, whilst the homebound ant has a clear destination, its progression along the path that leads to it is far from a straight line, due to the numerous obstacles encountered on the way. The example inspires in him this startling observation:

*An ant, viewed as a behaving system, is quite simple. The apparent complexity of its behavior over time is largely a reflection of the complexity of the environment in which it finds itself.*

More than fifty years after the first mention of the *parable of the ant*, the general question is still very much alive. There is indeed an ongoing debate today as to what aspects of the behaviour, and especially the social behaviour, observed in an animal species can be explained as a specific adaptation versus an emergent property of simple behavioural rules when individuals interact with their environment. If some emerging properties of a group's social structure result from more simple behavioural mechanisms, it may be that what is thought to be an explicitly social behaviour does not require specific selective adaptations.

Although animal groups often display astonishing emergent patterns in their collective behaviour [2], recent research has shown that much of the complexity of some natural phenomena can be directly attributed to the collective dynamics of simple, self-organised processes and individuals [3]–[5]. In recent years, assumptions of random behaviour have been discussed at length in contexts such as animal movement and foraging [6]. There have also been specific investigations on the description of collective motion in biological systems based on stochastic processes, as exemplified by the concept of *Brownian agents* [7], [8]. This framework has proven to be a versatile and practical approach to describe collective patterns of movement at different organisational levels, for systems ranging from bacteria [9] to crustaceans [10] and social insects [11]. However, clearly the behaviour of animal groups is not limited to collective motion, but also includes complex social interactions between individuals within the group. These interactions are often of a high-order and individually-differentiated nature, as illustrated by the long history of studying social relationships in animal species with high levels of cognitive development [12], [13]. Despite the efforts mentioned above to describe collective behaviour by means of stochastic forces, little attention has been paid to the importance of randomness for the emergence of complex social patterns in animal groups.

Here we use as a case study data from a population of wild house mice (*Mus domesticus*), social rodents characterised by cooperative breeding, polygynandry, territorial defence against non-group members, high skew in reproductive success and rather short mean life expectancy in both sexes [14]–[16]. This may have led to high flexibility in behaviour and social organisation. Yet exactly what aspects of the social structure of house mice can be explained by the self-emergent properties of collective random behaviour is unknown. In this paper, we first develop the assumption of random behaviour by creating a perceptual landscape that mixes purely diffusive motion and advective-diffusive Brownian bridges to describe the movement of individual animals. We then proceed to build a simple stochastic model that implements the assumed movement dynamics, and discuss its accuracy in accounting for specific characteristics of the social structure of a population of house mice. The novelty of our approach lies in the application of a dynamical perspective on habitat use to the investigation of social structure in animal groups as an emergent property of random interactions.

## Methods

### Ethics Statement

Animal experimentation was approved by the Swiss Animal Experimentation Commission (Kantonale Tierversuchskommission, no. 26/2002, 210/2003, 215/2006).

### Behavioural data

We study an established free-living population of wild house mice in a barn outside of Zurich, where mice can freely emigrate and immigrate. In the barn, the mice nest in artificial nest boxes, and are provided with straw as nesting material, and food and water outside the boxes. At four to six week intervals, comprehensive trapping is conducted to monitor the population, and adult mice are implanted with a transponder (RFID tag) so that they are individually identifiable. Transponder readers are installed in the tunnels that provide entrances to the nest boxes (two readers per nest box make it possible to distinguish a mouse that leaves a box from a mouse that enters a box); these readers connect to a computer and continuously track movements into and out of nest boxes. This provides 24-hour information on movements and social affiliations of adult mice (for a detailed description of the barn population and the methods used, see [17]). Data collection started in May 2007 and totals around 29 million individual recordings as of June 2012. Here we analyse the period ranging from Jan. 1, 2008 to Dec. 31, 2009.

**Figure 1:**
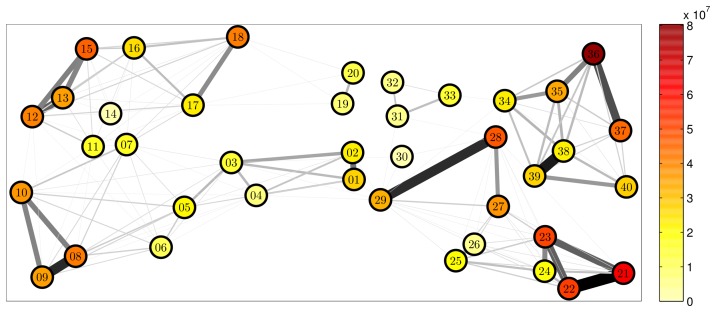
Box occupation pattern and traffic between nest boxes. The colour of the boxes represents their cumulated stay duration in seconds, whilst the darkness and thickness of the interbox edges represent the intensity of the bidirectional traffic between the boxes (for clarity, only edges with a traffic 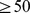 trips are displayed).

Our 2-year dataset covers 11'259'557 location records for 508 mice, accounting for 1'376'720 stay events in 40 nest boxes, and leading to 1'064'695 one-to-one encounters inside nest boxes, whose frequency, context and duration we use as a proxy for the characterisation of social interactions (the full Dataset S1 is available to download from the Supplementary Material page). Figure 1 shows the geographical positioning of the nextboxes, as well as the heterogeneity of their occupation pattern: indeed, the total occupation duration per box ranges from 264 to 22'332 hours (the lowest figure may, however, be attributed to a malfunctioning RFID antenna; the maximum value is longer than our study period because some stays can overlap when two or more mice stay simultaneously in the same nest box). This aggregated view allows to identify the “hot spots” of the barn and the busiest routes between nest boxes, from a static perspective on the behavioural data. It also shows how geographically clustered the traffic between nest boxes is, as a result of the partitioning and the obstacles of the barn. Evidently the physical environment of the mice affects their movement, which in turn has an impact on their social contact patterns. However, the view presented in Figure 1 is insufficient to characterise the dynamics of movement of individual mice, and the link between these dynamics and their social behaviour. Indeed, it focuses on aggregated properties of the study system rather than dynamical ones.

### Construction of a probabilistic landscape to describe animal movement

#### Objective of the approach

As Lima & Zollner put it [18], “we know remarkably little about the sorts of information available to animals at the scale of ecological landscapes, and we know even less about how such information is used in decisions regarding movement”. Our goal here is to use the movement dynamics (successive sampling events) of mice to reconstruct the perception they have of their environment, and create a landscape object describing this perception. We call this object perceptual landscape; it is shaped by the deviation of individual mice from a null assumption about their movement across the environment. The perceptual landscape approach departs from the static perspective presented above, and constitutes a null model to understand to what extent certain social patterns can be explained by the inter-independent movements of individual mice. In the following two sections, we detail the construction of the perceptual landscape. Note that, in addition to the technical details developed here, an abridged description of the complete method is presented in Table 1.

**Table 1:** Abridged summary of the perceptual landscape method, with references to the corresponding parts of this article.

**Rationale**	• Tool to map the environment of animals from their perspective rather than from the way we see it.
	• Can be used as a null model of social behaviour, to transfer the complexity of many interacting individuals to a single landscape object.
**Landscape construction**	1. Define a set of sampling locations (such as resting areas or nesting sites) and measure the time spent at those locations by an animal or a set of animals, as well as the transit time between two locations (illustrated in Figure 1).
	2. Construct a set of Brownian bridges between each pair of locations successively visited by an animal (Eqs. 1–9, Figure 2A). The shape (depth versus width) of each bridge will be defined by the difference between the average speed of the animal during that transit and its assumed maximum linear speed, i.e. its degree of meandering along the path.
	3. Define the attractiveness of the static locations based on the time spent at those locations by animals, and construct the corresponding “wells” in the landscape (Eqs. 10–12, Figure 2B–C). The deeper a well, the longer the periods spent at the corresponding location.
	4. Add both landscape components (Brownian bridges and wells) to obtain the full perceptual landscape (Eq. 13, Figure 3).
**Input data**	Animal location data (e.g. obtained from direct observation, GPS measurements, radio tag identification) and position of the sampling events, with a timestamp on each of the successive locations visited. Large sample sizes allow for a more accurate reconstruction of the landscape.
**Parameters**	The method presented here includes only one user-defined parameter: 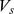 , the assumed moving speed of an animal during a transit between two locations. All the other parameters can be measured from the input data. Additionally, we detail (see last paragraph of the Methods section) a way to calibrate 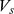 against the observation data, by assuming no diffusion (i.e. a straight trajectory) for the fastest measured transit.
**Output format**	A matrix of discretised height values for the landscape, describing either a three-dimensional structure (Figure 3A) or a heat map (Figure 3B–C).

#### Movement between sampling locations

Drawing from the now well-developed argument that the notion of animal home range is ill-defined when only derived from a static perspective on animal position [19]–[21], we use superimposed Brownian bridges [19] to characterise the expected spatial behaviour of an animal between two locations at which its position has been sampled.

Let us first start with the case of an animal being sampled consecutively at two different locations 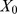 and 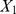 at times 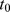 and 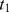, respectively. Without any further information on the nature of the motion between the two locations, we may only assume that the animal can be considered as a particle a constant drift 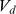, taking it from 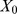 to 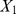 during the time 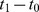. Let 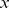 be an axis parallel to the general direction of motion, 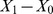, and 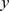 an axis perpendicular to 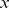.

With these assumptions, the motion of the animal can be described by a Langevin [22] equation,
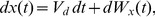
(1)

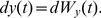
(2)The two independent Wiener processes 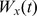 and 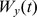 describe the diffusion process along both axes. We assume that the fluctuations of the particle are described by an isotropic diffusion coefficient 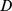. Thus, over a small time interval 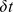, the increments of these processes, 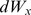 and 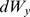, are drawn from a Gaussian distribution with mean 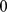 and variance 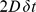. Because the increments are not correlated over time, these processes are completely defined by their mean and variance: 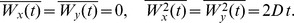


The expression of 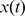 and 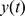 can be obtained by direct integration (see Text S1 from the Supplementary Material). At any time, the distance travelled from the initial point, 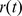, is simply given by 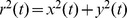. The fluctuations of the particle are described by a linear and isotropic diffusion coefficient 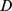. Under these conditions (for a detailed development of this derivation, refer to Text S1), the mean distance travelled at time 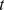 is given by
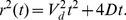
(3)


We now consider a single excursion in which the particle starts in 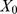 at 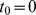 and ends in 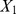 at 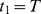, with a constant diffusion coefficient 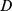. The transit time 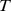 and the linear path length 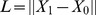 between the starting and ending locations allow us to estimate the drift coefficient as 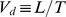. In the following, it is convenient to introduce an additional *effective velocity* measure for a transit. To define it, we first note that the animal has a motion that, whilst intrinsically fluctuating, does not reach the infinitely small granularity of an ideal random walk. We denote the average effective velocity of this motion as 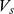. Thus, we can write upon arrival 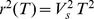, which together with Eq. (3) yields the following expression for the diffusion coefficient 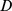 for this trip:
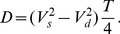
(4)


A Brownian bridge [19]–[21] describes the Brownian motion of a particle whose position is known at two different points in time. The assumption of a Brownian bridge to describe a transit between two successive locations is not strictly valid in the context of animal movement data, as it implies that the particle's position is known with absolute certainty at both ends of the bridge. Not only is this untrue practically when measuring the position of an animal, but it also induces diverging probability densities around the ends of the bridge. There is an error, inherent to location data, that must be accounted for [21]. We thus extend the usual definition of a Brownian bridge and assume that the variance 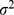 of the associated Wiener processes [23] in each direction has finite values at the start and the end of the bridge: 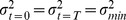. During the transit, the variance of the Wiener processes is given by the following time-dependent expression [19]:
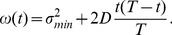
(5)This means that the variance of the expected position of the particle is maximum at half the transit time 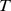. Along the Brownian bridge, the average position of the particle at time 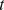 is simply 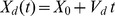, i.e. on average the particle moves towards the end of the bridge pushed by its constant drift 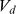.

We can now write the probability density function (PDF) of the particle's position 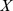 as a function of time:
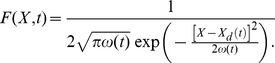
(6)

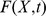
 is a time-dependent PDF. A time-independent representation of the density 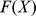 can be obtained through direct integration:
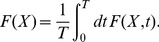
(7)

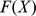
 is the static Brownian bridge representation of one transit between two locations. A set of bridges can be computed for all sampling events associated to one animal, a set of animals, or a whole population, depending on the application. In our case, we define the set of 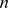 bridges connecting any pair of locations where an animal has ever been sampled (the 40 nest boxes). By summing up all these bridges, we obtain a global PDF defined on the whole domain studied (the complete barn):
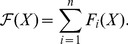
(8)

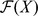
 is the global stationary occupancy function of the system. As a stationary PDF, it has an associated bivariate potential landscape 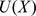, linked to it by the relation 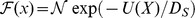 [22], with 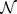 a normalisation constant for 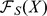 and 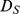 associated with the temperature of this newly-defined system (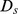 is independent from the 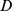 previously associated with each Brownian bridge). As 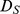 acts here simply as a scaling constant for the landscape, we can set 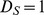 and obtain the canonical form of the landscape associated with this dynamical regime:
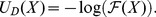
(9)


#### Exit from a nesting site

In a second step, we include in the landscape the influence of those periods in which the position of the animal is roughly constant, corresponding typically to a period of rest in a nest box. We further our previous comparison of the animal to a particle performing a stochastic motion, this time without adding the drift component. Its behaviour can thus be described by a purely diffusive motion.

**Figure 2:**
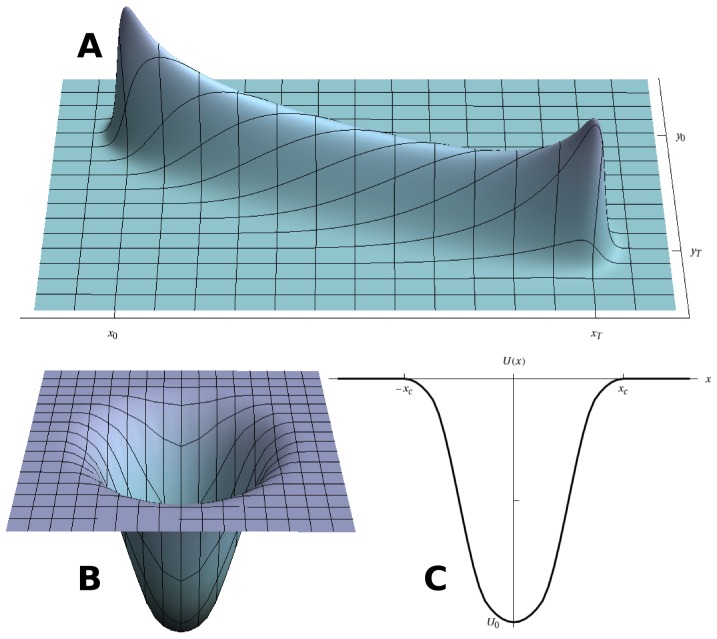
Brownian bridge and potential well used in the construction of the perceptual landscape. **A.** 3D representation of the probability density function (PDF) associated to a Brownian bridge starting in 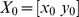 at 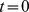 and ends in 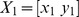 at 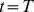. **B.** 3D representation of a potential well used to describe the motion of an animal when in a static location (here a nest box), and **C.** cross-section through the middle plane of this well, with 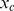 the radius of the well, and 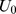 its maximum depth at 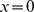 (its minimum depth being 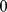 at 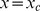). The complete well between the approximations near 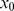 and 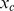 is constructed using a continuous approximation (here a spline), but its shape has little influence on the escape time of a particle from the well.

However, one needs also to consider that the nest box possesses a certain attraction, making it unlikely for the animal to exit it shortly after entering it, as would be the case for any other place visited when on the move. In this stochastic setting, these nesting sites can be described as wells of potential, with a circular boundary that the particle has to overcome to exit. In such a system the escape time – or mean first passage time (MFPT) of the particle through the boundary – 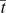 is well-known from theoretical studies on stochastic dynamics [24], [25]. We consider the simple case of a single-well paraboidal potential [24], with radius 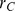 (see Figure 2). We set the escape potential 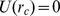, and represent the minimum potential by 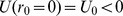. As discussed in [22], the escape time of the particle from the well is rather insensitive to the actual location of the boundary 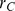, but depends mostly on the height 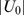 of the potential barrier, and the diffusion 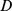 of the particle inside the well. This approximation holds whenever the particle diffuses fast enough in both directions, so that the escape time is governed by the time it takes the particle to climb the potential barrier.

Because of the radial symmetry of this system about the axis of the well, we focus now on the radial motion of the particle. The radial diffusion coefficient is given by the composite of the diffusion terms in both directions, i.e. 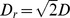. Writing the Taylor series approximation of 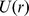 around 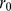 and 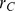 lets 
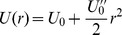
 near 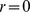 and 
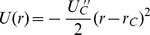
 near 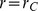, since in both points the first derivative of the potential is null: 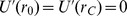. The escape time obtained by imposing a reflecting barrier at 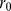 [24] is
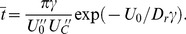
(10)In behavioural studies, what is generally measured is the time an animal spends at the nesting site or nest box, here 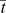. By setting 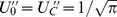 for simplicity, we derive from Eq. (10) the depth of the corresponding well in the landscape as
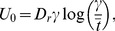
(11)where 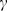 is the friction constant, which we can conveniently set to 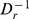, or use as a scaling constant for the height of the landscape. It should be noted that, whilst they have disappeared from Eq. 11, 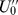 and 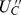 represent the curvature of a well at its bottom and boundary, and thus the scale of the landscape (set by the value of 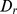) should be chosen accordingly to allow for a smooth shape of the well (for a small 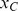, the curvature is high). The rest of the well can then be built from a continuous approximation (typically a spline).

**Figure 3:**
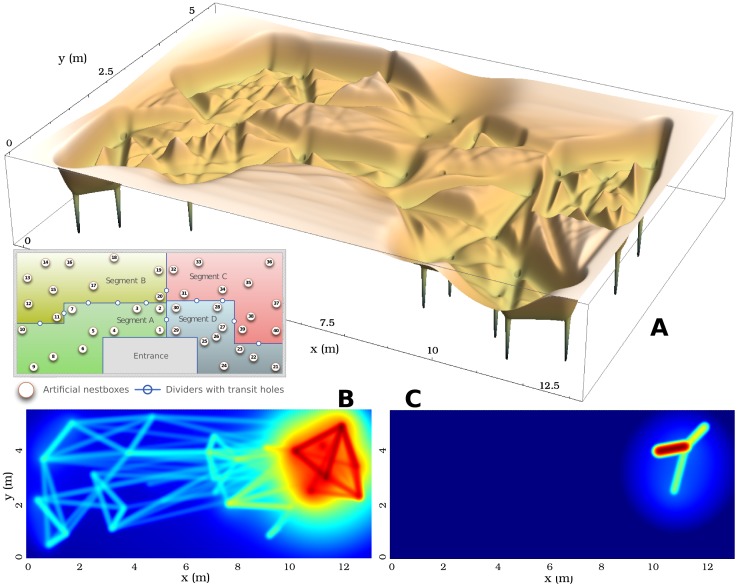
Perceptual lansdscape for both the complete population and specific individuals. **A.** 3D rendering of the perceptual landscape of the complete population of wild house mice, showing the discrepancies in occupation density of the different regions of the barn. The inlay is a schematic representation of the barn, with the disposition of the nest boxes and the dividers creating 4 artificial territories. Interestingly, this physical structure is represented accurately in the landscape, with high grounds following the dividers (except between segments A and B), and an elevated plateau around the entrance to the barn, isolated from the rest of the structure. Some of the wells, corresponding to each of the nest boxes, can be seen beneath the landscape. **B.** and **C.** The perceptual landscapes of a male (id 0006B8C03C) and a female (id 0006B9BAB9) with two very different patterns of spatial activity, displayed as temperature maps; red areas denote a low elevation of the landscape (higher probability of finding the animal), whilst blue areas correspond to higher regions of the landscape (lower probability); the color scaling is the same in both graphs. Despite the two mice having the same core areas (Segment C of the barn), their home ranges differ vastly in that the male concentrates its activity around 4 boxes only, whereas the female's home range extends well beyond this. It can be observed that most of the diffusive motion occurs within the core nest boxes, as opposed to more advective motion outside this area. This may hint to behavioural differences when roaming within or without an animal's territory (part of the home range that is defended). Schematic map of the barn courtesy of Rico Leuthold.

We have made here a spatially-explicit description of the well to allow for its inclusion in the three-dimensional landscape (see Figure 3). In spite of the number of parameters involved in this full spatial description, the construction of the well is fully governed by one parameter only, namely its depth 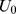, which can be obtained from the escape time 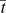. The construction of the complete well from the quadratic approximations is depicted in Figure 2. The global potential associated with the animal's nesting behaviour is then simply the sum of all the potential wells 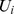 associated with individual resting locations 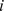,
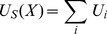
(12)Considering the motion of the particle within the well is equivalent to setting an absorbing barrier at 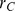, upon crossing of which the particle returns to a stochastic motion with added drift. This corresponds to the moment when the animal exits the nest box to travel towards a new location.

#### Perceptual landscape

The complete landscape is obtained by adding together (i) the dynamical landscape, extracted from the sum of all Brownian bridges and (ii) the landscape built from nesting patterns, based on all potential wells around static locations:
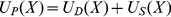
(13)This yields a global landscape 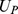 which derives from both the travelling and resting behaviours of the animals. This landscape is shaped by the reaction of animals to their environment: indeed, an accumulation of slowdowns or detours around a perceived obstacle will result in the creation of a raised “mound” in the landscape, and an area exterting particular attraction on the animals will be signaled by a lower dip or trench. The elements shaping the landscape include not only geographical features of the environment, but also any factor that the animals perceive and react to by modifying their movement (this is developped in the Discussion section). Therefore, the landscape effectively describes the perceived environment of the animal, hence the name of perceptual landscape. This is a generalisation of the notion of landscape of fear [26], [27], which implies that the home range of an animal depends to a great extent on the preying range of its predators and the availability of resources. The perceptual landscape includes not only the information the animal has about its resources and predators, but also everything that influences the way it moves about in its environment, such as social interactions with other individuals. This therefore constitutes an integrative tool to describe and analyse the movement of an animal and how this movement is influenced by its complete physical and social environment.

#### Application to the spatial dynamics of wild house mice

We apply the technique described above to represent the perceptual landscape of the population of wild house mice in our dataset. When building the Brownian bridges, 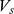 is an important parameter, as it scales the quantity of diffusion along any bridge. There is a rather straightforward method to set 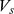, by assuming that there is no diffusion (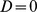) along the path where the drift speed is maximum. In the expression of 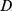 (Eq. 4), constraining 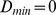 on the “straightest” bridge yields 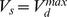, since 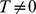. In other words, along the straightest Brownian bridge, we assume that the unique component of the motion is the drift. In this case, we compute 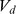 as the distance between both ends of any bridge divided by the mean transit time along this bridge (formally, the computation of the landscape should be carried out individually for each transit ever observed between two boxes. Because of the high computational costs involved in this approach, we compute the bridges using a unique value of 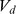). To avoid bias due to small sample sizes, we only consider those bridges that were crossed at least 50 times (10 times for the individual landscapes of Figure 3), which still leaves us with 532'969 crossings of 280 different bridges. Then we set 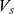 as the maximum value of all 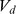, so that the bridge that was crossed on average the fastest is considered to have been crossed in a straight line. Here 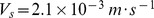 (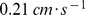), which may seem like a rather low value but illustrates the fact that mice spend a great part of their time not moving (developed in the Discussion section). The distribution of transit times from a box to another is highly skewed and heavy-tailed (see Figure S3), which gives little significance to its mean [28]. Therefore, when creating the Brownian bridge between any two boxes we use the median rather than the mean of all transit times to mitigate the influence of very large (and rare) transit times. Following this, each bridge 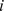 is associated with a different diffusion coefficient 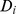 and a frequency of occurrence 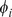. In order to reflect this general diffusive behaviour on the nesting landscape (corresponding to the periods in which the mice stay inside nest boxes), we use the weighted average 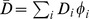 of all dynamic diffusion coefficients and use it to calculate the corresponding radial diffusion coefficient 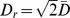. This, added to the known average leaving time from each nest box, allows us to compute the depth of each corresponding potential well. As the nest boxes have a diameter of 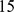 cm, we set 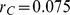 m. The resulting perceptual landscape, both for the whole population or for individual mice (Figure 3), yields new insights into the way the animals perceive their environment.

## Results

### Multi-agent implementation of the perceptual landscape model

Through the description of the perceptual landscape, we have developed the assumption of simplistic individual motion to reconstruct the environment of a wild house mouse. We now test this hypothesis by implementing the assumptions of the perceptual landscape in an elementary model of collective behaviour, in which all agents are governed by the principles of random motion we have introduced previously. We make the assumption that through the collective behaviour of those agents, whose complexity lies far below that of real mice, we can reproduce some of the global behavioural patterns we observe in the barn.

#### Model selection

The most intuitive way of implementing the perceptual landscape's assumptions would be to construct a complete diffusion model, in which the movement of the agents across the landscape is governed by the rules presented above. However, the data set against which we are testing this model contains information on the social interaction of mice inside nest boxes only. Another equivalent (in this context), more parsimonious modelling assumption thus comes to mind: a simpler description of the collective dynamics of the stays in nest boxes and the transitions between them, which can be fully described as a set of simple stochastic processes, or Markov processes. Because we simulate these dynamics across the landscape associated to the whole mouse population, this approach amounts to discarding interindividual differences and simulating the behaviour of many identical agents. Each of these agents is a blend of all real individuals, and therefore represents the “average mouse” from the study population. The following section describes the implementation of this parsimonious modelling assumption and its calibration based on the experimental data. For illustrative purposes, a simulation of the complete diffusion process across the perceptual landscape is also shown in Video S1.

#### Agent-based simulation of the stochastic model

We use a standard stochastic simulation technique known as the Gillespie algorithm, [8] defined as follows: the system is composed of 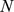 agents, each characterised by their current state (inside a nest box or travelling between two nest boxes), and their own transition time 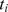 and transition rate 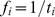. We define the system's mean transition rate as 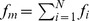. This is the mean frequency at which the system is expected to change (i.e. an agent in the system either enters or leaves a nest box). The mean transition time is 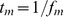. The simulation time step 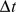 is a random variable which depends on the current state of all agents: for each iteration, the current time step is sampled from an exponential distribution with mean value 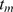, so that 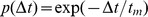. This fully represents the stochastic dynamics of stays in nest boxes and the duration of interbox transits which we have assumed in the construction of the perceptual landscape.

Let 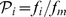 be the probability that agent 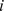 changes state (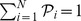). We then select the agent 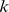 whose state changes by mapping the set of transition probabilities 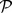 to a uniform distribution, i.e.
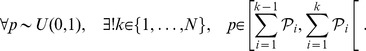
(14)In other terms, 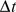 indicates when a change in the system happens and 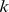 indicates what this change is, according to the transition probabilities of each agent. In order to run the simulations, we set a constant number of agents in the system, equal to the average number of mice detected per day at the barn. We obtain the leaving and transition rates as the inverse of the average leaving and transition times (extracted from the data) from each box to each other box. For sampling reasons (see Eq. 14), we normalise those matrices so that their sum is 1. We initially distribute the agents according to the occupation rate (inside or outside a box) and the occupation preferences (described in Figure 1). The simulation time range is set to a period of two years, the same period as that covered by the empirical dataset.

**Figure 4:**
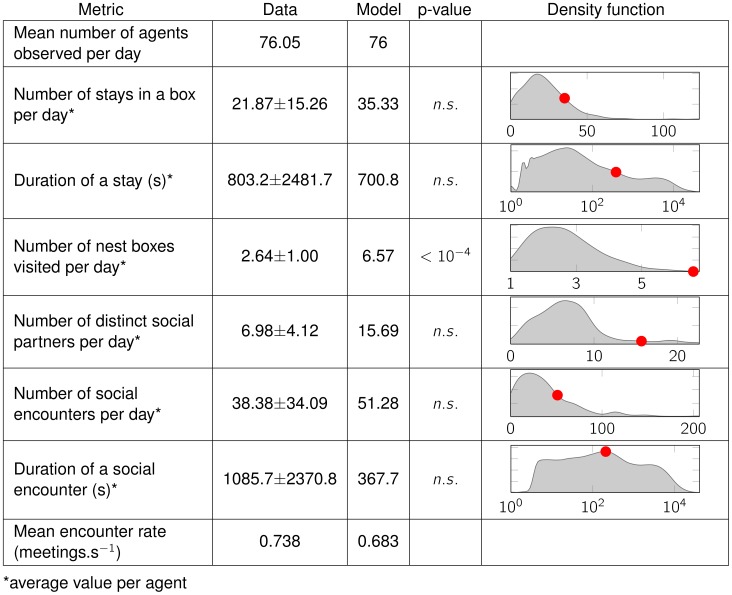
Results of the multi-agent simulation compared to metrics from the real population. Comparison of some behavioural metrics between the experimental data and the simulation output. The experimental data is given as mean 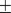 standard deviation of the observations, the simulated data as mean only (justification in the text). The 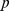-value given is that of a bootstrapping analysis to determine whether the mean value of the simulated metric may fall within the 95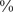 confidence interval of the experimental distribution. These results are illustrated by the graph of the density function estimate (smoothed using a Gaussian kernel) of the experimental distribution over which is superimposed (red dot) the mean value of the simulation output. The mean number of agents in the system and mean encounter rate are single values, therefore no standard deviation or density function is given. The encounter rate is computed by dividing the total ratio encounters/stays by the average duration of a stay, giving an indicator or the social “activity” of the system as a whole.

The aggregated results of the agent-based simulation are in very good agreement with the aggregated behavioural data. Indeed, the correlation between real and simulated occupation rates and transit counts is very high (Pearson's correlation coefficients 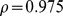, 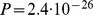 and 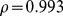, 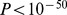). This result is developed analytically in the Supplementary Material (Text S1) by developing a detailed Markov chain description of the model. Beyond this stationary perspective, we compare the experimental and modelling results for other metrics pertaining to the behavioural dynamics of the agents, by testing whether the mean value of the distribution of a synthetic metric may come from the distribution of the corresponding one in the experimental data, as summarised in Figure 4. This is proper practice as all metrics considered in this table for synthetic data can be grouped in two categories. The first category comprises the duration of a stay in a nest box and the duration of a social contact, which are exponentially distributed according to the model rules. All the other metrics belong to the second category, and are normally distributed (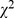-test, 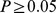 always) with a variance much lower than that of their experimental counterparts. Therefore, the distributions of the synthetic metrics can simply be described by their sample mean. We compare these mean values to the distribution of experimental values by using a bootstrapping approach [29], under the null hypothesis that the sample mean of the synthetic metric belongs to the 95 central percentiles (95% confidence interval) of the experimental distribution. Remarkably, we find that our stochastic assumption produces results which are not significantly different from the distribution of real values; this is especially important when considering those metrics that pertain to social behaviour, such as meeting duration or number of social partners per day. There is however one factor that the model significantly underestimates, namely the territorial behaviour of the mice (expressed in the number of nest boxes visited per day).

## Discussion

### The perceptual landscape as a novel method to map habitat use

In this paper we have developed the assumption of simple stochastic processes as a driving force for social interaction in a population of wild house mice. We introduced the notion of perceptual landscape, which maps the patterns of movement of mice between nest boxes in a barn into the motion of stochastic particles within a potential field. We are well aware that our model ignores the fact that such patterns have resulted from natural selection and adapt mice to their environment. Instead, we are interested in analysing whether and to what degree a general movement pattern alone can reflect important characteristics of the spatial and social behaviour of free-living mice.

Our approach integrates two important facts, often neglected when mapping the home range or territory of wild animals: (i) the movement from one sampled location to another ought not to be thought of as a straight line, but instead may be better approximated by planar diffusion, and (ii) when an animal is resting in a safe area like a nest box (or generally visiting an area with a strong potential of attraction) it is less likely to exit and move on than if it were at another point within its home range. The parameters defining the landscape were obtained from the recorded data. The method has only one user-defined parameter, namely the assumed travelling speed of an animal. However, we detail a way to obtain this parameter from the data. Here we assumed a constant speed 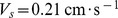 for a mouse moving along a Brownian bridge between two nest boxes. This may seem rather slow but is based on the fact that house mice spend a considerable part of their time outside nest boxes not moving, but instead feeding/drinking, marking their territory or partaking in social activities or territorial defence. Moreover, the mean radial diffusion coefficient 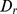 obtained from this value of 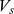 is 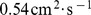; this amounts to exploring an area of slightly less than one centimeter per second in each direction, which is a sensible figure in the case of small animals like house mice. As a tool to study animal behaviour, the perceptual landscape method scales linearly with the number of active paths, i.e. pairs of locations with a large number of transits. Therefore we argue that the method could scale properly to much larger systems, and provide a new way to analyse the spatial behaviour of animals on a large scale.

### Behavioural implications

Interestingly, the perceptual landscape contains several of the structural features observed in the real landscape of the mice. This confirms previous observations [30], [31] that house mice use these structures to build up their own representation of the environment and navigate across it. From a formal point of view, it also reveals that the assumptions we made on the movement of random particles across the landscape yield meaningful behavioural patterns. Indeed, these patterns integrate important aspects of the decision rules guiding mice when they use their environment and defend their territory. Figure 3 illustrates the use of such a perspective for the study of individual home ranges (panels B and C), as well as the movement patterns of the whole population studied (panel A). We observed that the perceptual landscape is similar, but not identical, to the physical environment of the real mice. For example, the perceptual “wall” corresponding to the divider between segments A and B of the mouse barn is only weakly expressed. This may indicate that some mice regularly use nest boxes on both sides of the divider, and travel between them. Conversely to some physical features of the environment disappearing, some are overly expressed. For example, the entrance to the barn (see inlay of Figure 3A) is an area that mice could use, as it is open, but that they tend to avoid due to the presence of experimental equipment and the absence of protected nesting sites. As a result, the whole area appears in the perceptual landscape as a raised plateau, demonstrating little effective utilisation of that space. Generally, such discrepancies between the perceptual and the physical landscape may result from differences in the micro-environment of the animals. In practice, the mouse barn is not a homogeneous environment, but differs locally in humidity and temperature, in the degree of protection perceived by the mice (suitable hides or other spatial structures outside the nest boxes), in the exposure to popular traffic routes used by many individuals, in the availability of food and water in close proximity to a nest box, or in the amount of light (mice tend to avoid bright areas [14]), etc. In addition, the movement of mice between nest boxes will be influenced by their social environment. Since the perceptual landscape integrates all such factors, it may be seen as a cartographic tool of a much higher precision than a standard schematics or map of the environment of an animal population. In other words, this landscape is the combination of all dimensions that animals perceive in their environment (be them physical boundaries, temperature, humidity or presence of conspecifics). This is especially important when considering that many animals view their environment in a way that is different from the way we see it. Indeed, house mice have poor visual acuity and their world is “dominated by smell” [14]. The representation of an animal's environment by simply mapping it as we would map our environment may thus be misleading.

### Extension to an adaptive framework

It is interesting to note that the perceptual landscape we described in this paper is a static construction, which represents the collective behaviour of the animals from a quasi-stationary perspective. As such, it results from the aggregate behaviour of the individuals of a population rather than adapts to it. However, the construction of this landscape by the animals is arguably a dynamical process: house mice, for example, alter their home range in response to the nearby presence of social partners [31]. In order to account for this dynamical aspect of the formation of the landscape, the Brownian agent framework [7], [8] may be more suitable than simpler stochastic particles: indeed, Brownian agents move across an *adaptive* landscape, which builds up over time as a result of their behaviour. The Brownian agent paradigm constitutes a natural framework to study non-equilibrium systems, such as a population of interacting individuals. As such, this framework could be a logical extension to the perceptual landscape technique.

### Individual complexity versus emergent social structure in our mouse population

In a second step, we tested the accuracy of the assumptions of our landscape. To this purpose, we implemented an agent-based model of the behaviour based on the landscape assumptions and tested its results against real observational data. As we assumed a very simple individual behaviour (stochastic motion) for the mice moving across the landscape, a simple approach was sufficient to reproduce it. We described the transitions between nest boxes by a set of stochastic processes, or zero-order Markov processes, effectively representing the set of nest boxes as a Markov chain (this comparison is developed in Text S1). In this description, the escape rates from each state (inside a nest box or moving between two nest boxes) were calculated from the real aggregated data. The underlying assumption of this Markov approach is that the process has no memory, the transition probability being only dependent on the current state. In this paradigm, individual mice are particles travelling along the Markov chain and all follow the same rules of motion. Each particle can be thought of as representing the “average mouse”, an individual who behaves as a composite of all the mice from the real population, without particular individual characteristics. We aimed to study how such an approximation performs in a social context, or how well it may reproduce the observed patterns of social encounters in a wild house mouse population. We used in our simulation as many average mice as the average number of RFID-tagged mice in the barn over the two-year study period. It should be noted that this approach implies no *a priori* assumption on the importance of the social interactions that can occur each time two mice meet inside or outside of a nest box, although it is well-known that social aspects play a crucial role in the behaviour of house mice, especially female [30], [32]–[35].

Remarkably, we found that this simplistic, randow walk-like approach is sufficient to reproduce some features of the nest box occupation patterns, both at the population and the individual levels. In other words, the collective dynamics of the population as a whole, with its intrinsic fluctuations (birth and death cycle) and interindividual differences, may be well approximated by the behaviour of the average mouse. This is obvious from the match between experimental and simulated data in occupation density and transit frequencies between nest boxes. This accurate match should, however, come as little surprise: indeed, these features are aggregated observations on the behaviour of the whole population, and precisely those whose estimate we used to calibrate the stochastic model. Of more interest is the comparison between the model output and the observational data with regard to higher-level social features. The statistical test we ran on the simulation results amounts to asking whether the average mouse, moving randomly across the perceptual landscape, has a social behaviour (defined as its pattern of encounters inside nest boxes) consistent with the social behaviour of a real mouse from our study population. In agreement with the results obtained at the population level, we found that at the individual level most of the social features in the average mouse's behaviour were compatible with the behaviour of a real mouse, with the exception of the territorial aspect (expressed in the number of nest boxes used per day). This is especially interesting when considering that we excluded any influence of conspecifics on an individual's probability to enter and/or stay in a nest box. Yet, the patterns of social interaction (number of social encounters per individual, number of social partners, or duration of a social encounter) did not differ significantly from those observed in the population of real house mice. It is important to note, however, that mice tagged in the study population are only adults, which typically had already established their territory and integrated in a social group. The behaviour of young, dispersing individuals that still move between groups or territories is thus underrepresented, although it may differ. Once integrated in a social group, however, mice seem to regularly meet with all group members, without pronounced individual preferences. Nevertheless, many real mice had fewer social partners and fewer social encounters than the average mouse from our model (although the differences were not statistically significant from the global population). These discrepancies may, again, reflect territoriality, social preferences and differences in reproductive dominance [17]. Indeed, from a behavioural perspective, the assumption made for the average mouse, whose behaviour is the average of that of all other conspecifics, explicitly ignores any variability among individuals. However, it is well established – not only for house mice – that individuals within species and populations vary in their behaviour according to their sex, age, dominance or reproductive status, or personality. Even within individuals, behavioural performances can change over time due to individual experiences and modifications in physiology (hunger, hormones, etc.). All such individual variability can explain differences in competitiveness, aggressiveness, social tolerance, or boldness, which will affect individual preferences towards conspecifics as well as towards nest boxes or other spatial structures. Furthermore, hindering non-group members from entering own nest boxes is very important due to the tendency of mice of both sexes to kill non-offspring. Evidently, the omission of all such factors in the model leads to different movement and social interaction patterns, changing the structure of the social network. Of course, it is highly implausible that a random particle may faithfully mimic a living mouse, and the simple modelling approach we have presented could not pretend to fully reproduce the complex, dynamical social network of a population of wild house mice. Yet, it points to the fundamental importance of simple, universal processes in the establishment of such a structure, and generally shows that the collective dynamics of stochastic processes are sufficient to reproduce some properties of a social system. This is clearly encouraging in the search for a more advanced cross-species model that could lead to a broader understanding of animal sociality.

We proposed the perceptual landscape as a framework in which the complexity of the individual interactions is transferred to that of the environment (the landscape). In doing so, we effectively simplified the analysis of collective social behaviour by moving from the study of many interacting individuals to the study of a single landscape object, whose properties can be quantified against those of the external environment (such as its physical structure). Moreover, this approach provided us with a null model for the social behaviour of the individuals in the landscape, which we could use to characterise the social network built by a population of average mice. In this regard, the results of the simulations presented in Figure 4 represent a good null assumption for sociality in animal groups. Notwithstanding the apparent performance of such a simplifying approach, it should be noted that there are key differences at the individual level between the average mouse and an individual from the real population. This points to the existence of more sophisticated rules governing animal behaviour than merely random principles, as can be expected from complex creatures such as social mammals.

We have applied methods from statistical physics to the understanding of the randomness underlying seemingly complex spatial and social animal behaviour. It appears that at least some elements of animal social behaviour can emerge from the collective dynamics of independent random processes. This complements recent work [36] which showed that such methods can be efficiently used to study the emergence of territoriality in animal populations. Such findings ultimately may parallel other examples of self-organisation in contexts as diverse as evolution [37], speciation [38] and even human economic behaviour [39].

## Supporting Information

Dataset S1 Full experimental dataset; for more information, refer to the complete description provided in the corresponding document included with the archive. The archive file can be downloaded at http://datadryad.org/handle/10255/dryad.43636 (doi:10.5061/dryad.c2b53).
(PDF)

Figure S1Markov chain model: diagram of the states an agent can occupy in the case where the number of nest boxes 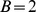. 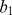 and 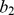 are the 2 nest boxes, to which are associated 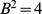 transit boxes corresponding to the intermediary states between any box and any other box (including itself). Edges are labeled with the transition rates from state to state. The dashed lines represent the additional transitions from any state 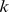 back to the same state.(TIFF)

Figure S2 Box occupation pattern and traffic between nest boxes obtained from a 2-year long simulation of the Markov chain model, closely matching the pattern observed in Figure 1 (same caption applies).
(TIFF)

Figure S3 Probability density function (PDF) of the distribution of transit times from any box to any other. Due to the high frequency of extreme events (very long transit times), the absolute mean of the distribution does not carry much meaning and we use the median instead.
(TIFF)

Table S1Comparison of the experimental average occupation density in the 40 nest boxes with the corresponding computed values from an initial distribution where all the density is concentrated in box 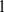, after a time 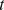. 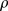 is the value of the Pearson's correlation coefficient between the measured occupation density vector and the stationary one, 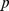 is the corresponding 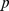-value in a two-sample Kolmogorov-Smirnov test under the assumption that the two distributions (instantaneous at time 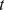 and stationary) of occupation density are different.(PDF)

Text S1 Full derivation of the expression for the mean square displacement of a particle. Additional information on the Markov chain model and analytical approach to its stationary distribution.
(PDF)

Video S1 3D representation of the perceptual landscape of the full study population (76 “average mice”), with a visualisation of their movement and interaction patterns. Yellow-filled dots are individual mice and grey empty circles are nest boxes, overlaid onto a 2D map of the landscape. The simulation is visualised with a sped-up time scale, with one second in the video representing five minutes of actual movement. The video is encoded using the H.264 codec and can be used with tools player such as VLC media player (http://www.videolan.org/vlc).
(MP4)
