# Supplementary material for: How Random Is Social Behaviour? Disentangling Social Complexity through the Study of a Wild House Mouse Population
Source: PLoS Comput Biol. 2012 Nov 29;8(11):e1002786. doi: 10.1371/journal.pcbi.1002786 (PMC3510074; doi:10.1371/journal.pcbi.1002786)
Supplement: Dataset S1 — Full experimental dataset; for more information, refer to the complete description provided in the corresponding document included with the archive. The archive file can be downloaded at http://datadryad.org/handle/10255/dryad.43636 (doi:10.5061/dryad.c2b53). (PDF) [file pcbi.1002786.s001.pdf]

Introduction

This document describes the structure of the data set accompanying the manuscript [1]. It is partly adapted from [2]. The idea underlying this research project is to identify mice carrying an RFID transponder at specific locations. The locations where this identification occurs are the 40 artificial nest boxes distributed in the barn (see full description in [1]).
An artificial nest box consists of a plastic cylinder (diameter and height about 15 cm) and a transparent entry tube, (20 to 25 cm long). Wrapped around the tube are two antennas with a coverage radius of 10 to 12 cm, capable of reading out the RFID transponders. Each antenna can be identified by an address which is set manually. To improve the antenna accuracy, the entry tube is bent to slow down the mice passing through. Fig. 1 depicts a model of such an artificial nest box.

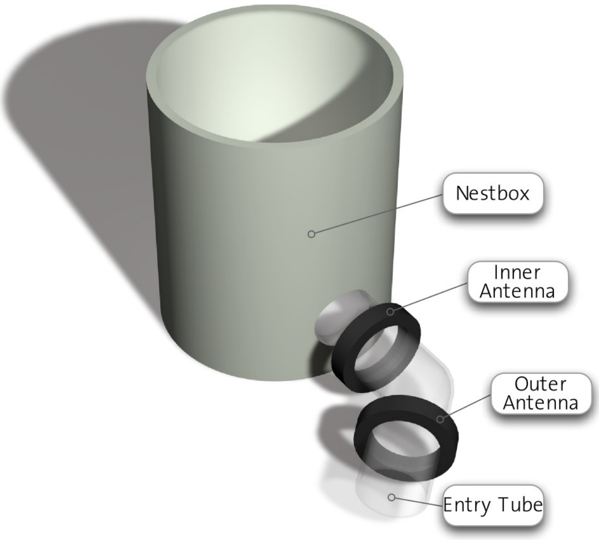

Figure 1. Rendering of an artificial nest box with the two antennas wrapped around the entry tube.

The RFID transponders used in mice are passive, i.e. they do not include a battery. The antenna acts as a scanner, presenting an inductive field that excites the transponder within antenna range. This energy is used by the transponder to send its identification to the antenna. Whenever a mouse carrying a transponder enters the coverage of an antenna, its identification is recorded and sent to a central computer along with the antenna address. The computer then adds a time value to the received data before writing it to a text file. The structure of these text files is detailed below.

Log files and format

The data set comprises 703 log files, grouped into yearly and monthly folders. The name of each file is a time stamp (yyyymmdd_HHMMSS.txt) representing the time at which the collection of the data logged in this file started. The maximum range covered by a single file is 24 hours, as the logging program automatically creates a new file after 24 hours. In some cases, the system had to be restarted or was shut down for maintenance for a few hours, and the corresponding log file contains less than 24 hours of data.

The data files are simple text files, in which each line describes an event registered by an antenna in the system. There are two types of events:

- The first type occurs if the antenna could identify a transponder. In such a case a line as shown in Fig. 2 is written to the data file. The ID of the transponder is a unique, ten-character value.
- The second type of event, for which the resulting data line looks as shown in Fig. 3, occurs when a transponder enters or leaves the coverage area of an antenna.

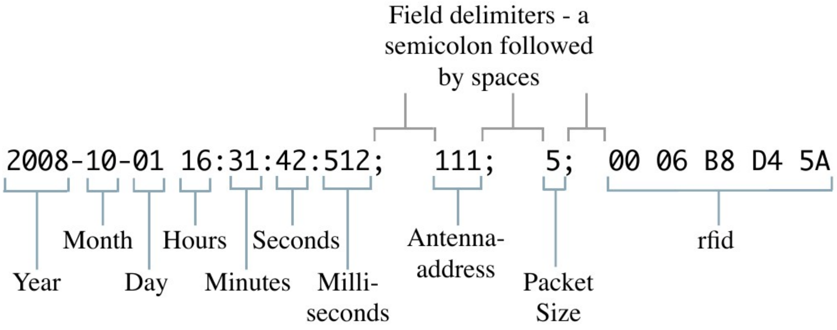

Figure 2. Typical dataset in a data file including an RFID transponder identification.

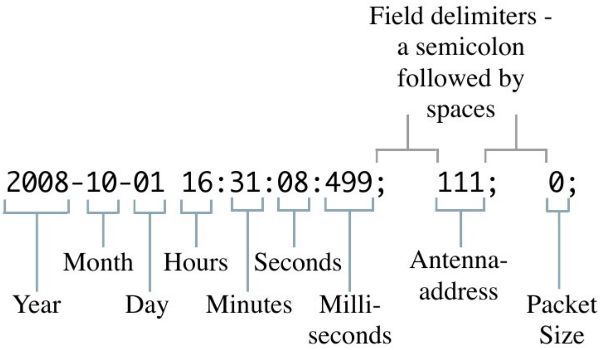

Figure 3. Typical dataset in a data file without an RFID transponder identification.

The following clipping and corresponding list show the data generated when a transponder enters or exists a nest box. The events are explained in the subsequent list.

```
1  2008—10—01  16:31:08:499;   111;   0;
2  2008—10—01  16:31:09:095;   113;   0;
3  2008—10—01  16:31:42:512;   111;   5;   00 06 B8 D4 5A
4  2008—10—01  16:31:42:807;   113;   5;   00 06 B8 D4 5A
5  2008—10—01  16:31:43:619;   111;   0;
6  2008—10—01  16:31:44:014;   113;   0;
```

The list numbering matches the line numbering of the clipping.

1. Transponder **enters coverage area** of the antenna with address 111.
2. Transponder **enters coverage area** of the antenna with address 113.
3. Transponder is **identified** as 00 06 B8 D4 5A at antenna with address 111.
4. Transponder is **identified** as 00 06 B8 D4 5A at antenna with address 113.
5. Transponder **leaves coverage area** of the antenna with address 111.
6. Transponder **leaves coverage area** of the antenna with address 113.

Depending on the event type, the value of the packet size is either a 0, for events without a transponder identification value, or a 5 if the transponder has been identified.

Antenna addressing

The antenna address is three digits long, composed of the box number it is attached to (first two digits) and the position at the entry tube of the box. Outer antennas have a 1 as the last digit of the address, inner antennas a 3 (e.g the antennas attached to box 11 are addressed 111 for the outer, 113 for the inner antenna, respectively). Box and antenna identifiers are unique. Due to technical problems, two nest boxes had to be renamed. These are:

- the box identified as "1A" (antennas 1A1 and 1A3) is in fact box 16.
- the box identified as "2A" (antennas 2A1 and 2A3) is in fact box 17.

References

[1] Perony, N., Tessone, C.J., König, B., Schweitzer, F. (2012). How Random Is Social Behaviour? Disentangling the Social Complexity of a Wild House Mouse Population. Submitted to PloS Computational Biology.
[2] Leuthold, R. (2009). A Data Mining Tool to Explore Social Behavior in House Mice. Masters thesis, University of Zurich.
