# Supplementary figures and images for: How Random Is Social Behaviour? Disentangling Social Complexity through the Study of a Wild House Mouse Population
Source: PLoS Comput Biol. 2012 Nov 29;8(11):e1002786. doi: 10.1371/journal.pcbi.1002786 (PMC3510074; doi:10.1371/journal.pcbi.1002786)

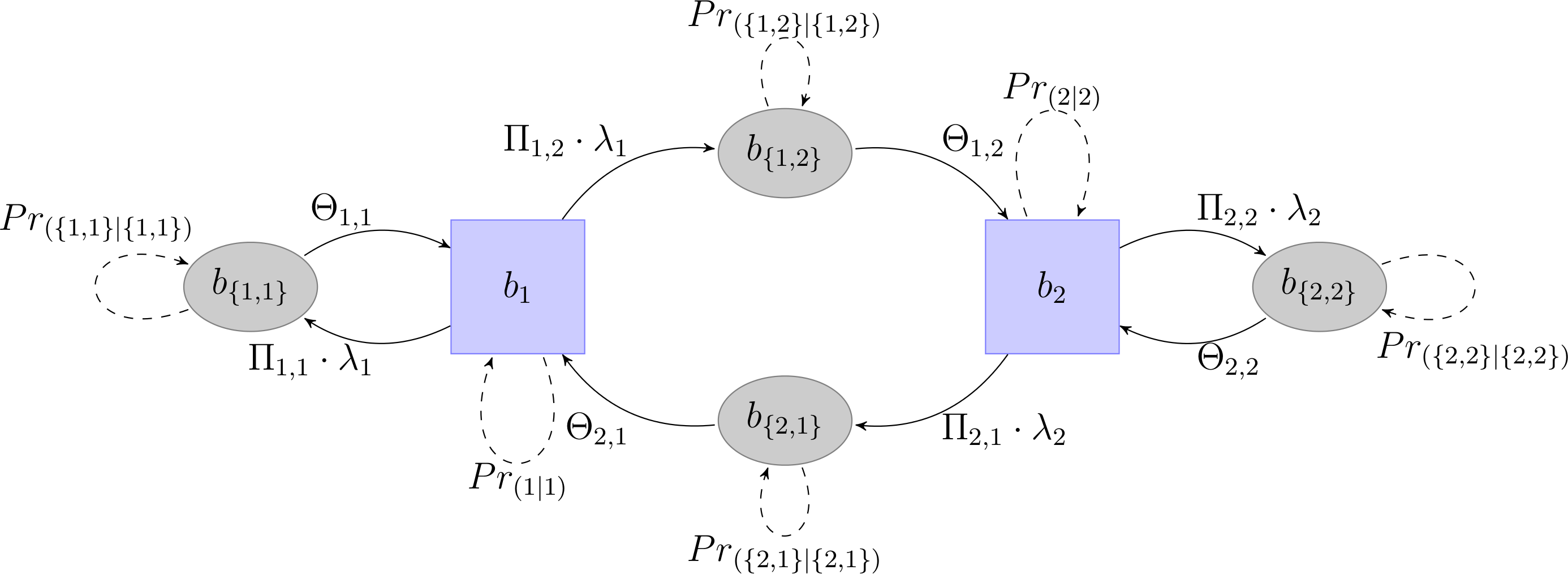

Supplement: Figure S1 — Markov chain model: diagram of the states an agent can occupy in the case where the number of nest boxes . and are the 2 nest boxes, to which are associated transit boxes corresponding to the intermediary states between any box and any other box (including itself). Edges are labeled with the transition rates from state to state. The dashed lines represent the additional transitions from any state back to the same state. (TIFF) [file pcbi.1002786.s002.tiff]

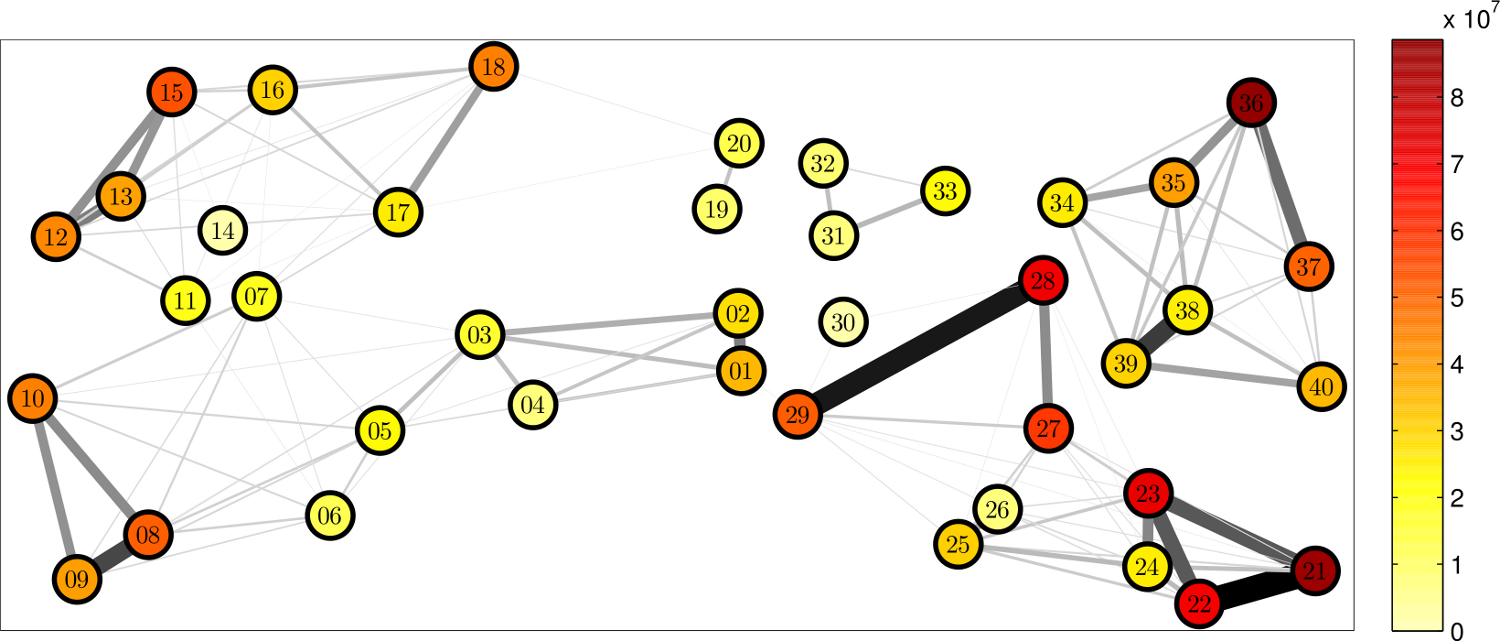

Supplement: Figure S2 — Box occupation pattern and traffic between nest boxes obtained from a 2-year long simulation of the Markov chain model, closely matching the pattern observed in Figure 1 (same caption applies). (TIFF) [file pcbi.1002786.s003.tiff]

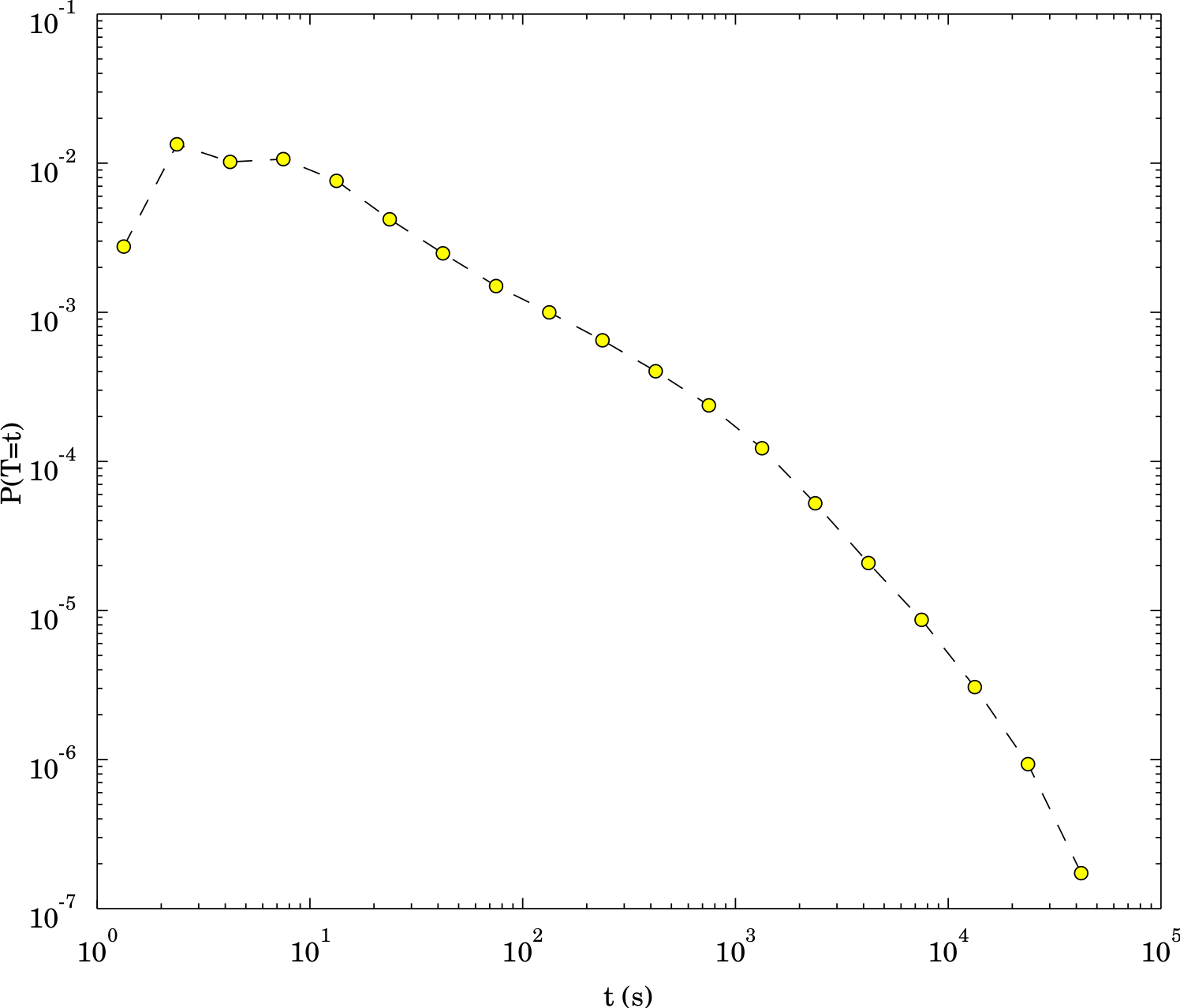

Supplement: Figure S3 — Probability density function (PDF) of the distribution of transit times from any box to any other. Due to the high frequency of extreme events (very long transit times), the absolute mean of the distribution does not carry much meaning and we use the median instead. (TIFF) [file pcbi.1002786.s004.tiff]
