# Supplementary material for: How Random Is Social Behaviour? Disentangling Social Complexity through the Study of a Wild House Mouse Population
Source: PLoS Comput Biol. 2012 Nov 29;8(11):e1002786. doi: 10.1371/journal.pcbi.1002786 (PMC3510074; doi:10.1371/journal.pcbi.1002786)
Supplement: Table S1 — Comparison of the experimental average occupation density in the 40 nest boxes with the corresponding computed values from an initial distribution where all the density is concentrated in box , after a time . is the value of the Pearson's correlation coefficient between the measured occupation density vector and the stationary one, is the corresponding -value in a two-sample Kolmogorov-Smirnov test under the assumption that the two distributions (instantaneous at time and stationary) of occupation density are different. (PDF) [file pcbi.1002786.s005.pdf]

How Random Is Social Behaviour?
Disentangling Social Complexity through the Study of a Wild House Mouse Population

Supplementary Table

t	ρ	P
1 hour	-0.0428	0.793
1 day	0.1778	0.272
1 week	0.7248	$1.2 \cdot 10^{-7}$
1 month	0.9972	$2 \cdot 10^{-44}$
6 months	0.9995	$< 10^{-50}$
1 year	0.9995	$< 10^{-50}$

Table S1. Comparison of the experimental average occupation density in the 40 nest boxes with the corresponding computed values from an initial distribution where all the density is concentrated in box 1, after a time t. ρ is the value of the Pearson's correlation coefficient between the measured occupation density vector and the stationary one, p is the corresponding p-value in a two-sample Kolmogorov-Smirnov test under the assumption that the two distributions (instantaneous at time t and stationary) of occupation density are different.
