# Supplementary material for: How Random Is Social Behaviour? Disentangling Social Complexity through the Study of a Wild House Mouse Population
Source: PLoS Comput Biol. 2012 Nov 29;8(11):e1002786. doi: 10.1371/journal.pcbi.1002786 (PMC3510074; doi:10.1371/journal.pcbi.1002786)
Supplement: Text S1 — Full derivation of the expression for the mean square displacement of a particle. Additional information on the Markov chain model and analytical approach to its stationary distribution. (PDF) [file pcbi.1002786.s006.pdf]

How Random Is Social Behaviour?
Disentangling Social Complexity through the Study of a Wild House Mouse Population

Text S1

Derivation of the mean square displacement

In this section, we derive the expected mean square displacement of an animal, considered here as a simple stochastic particle, as a function of time. The particle is assumed to perform a two-dimensional Brownian motion with an added constant drift V_d, which takes it from $\mathbf{X}_0$ to $\mathbf{X}_1$ during the time interval $t_1 - t_0$. $\mathbf{X}_1 - \mathbf{X}_0$, and y an axis perpendicular to x. At any time, the distance travelled from the initial point, $r(t)$, is simply given by $r^2(t) = x^2(t) + y^2(t)$. The drift V_d has a non-zero component along the longitudinal axis only. The fluctuations of the particle are described by a linear diffusion coefficient D, isotropic in both directions ($D_x = D_y$).

Under these assumptions, the motion of the animal can be described by

$$dx(t) = V_d\, dt + dW_x(t), \quad (1)$$

$$dy(t) = dW_y(t). \quad (2)$$

The two independent Wiener processes [a] $W_x(t)$ and $W_y(t)$ describe the diffusion process along both axes. Over a small time interval δt, the increments of these processes, dW_x and dW_y, are drawn from a Gaussian distribution with mean 0 and variance $\sigma^2 = 2D\,\delta t$. Moreover, the increments are not correlated over time. These processes are thus completely defined by their mean and variance:

$$\overline{W_x(t)} = \overline{W_y(t)} = 0, \qquad \overline{W_x^2(t)} = \overline{W_y^2(t)} = 2D\,t.$$

For one realisation of the processes $W_x(t)$ and $W_y(t)$, the position of the particle at time t, is obtained by direct integration of Eqs. (1-2),

$$x(t) = V_d \int_0^t dt + \int_0^t dW_x = V_d\, t + \int_0^t dW_x,$$

$$y(t) = \int_0^t dW_y.$$

Given that the Wiener processes have zero mean, the expected position after many realisations is then $\overline{x(t)} = V_d\, t$, $\overline{y(t)} = 0$. This calculation, however, does not contain information on the actual length of the path travelled by the particle, which we are interested to calculate since we want to quantify the effect of the stochastic fluctuations on the arrival time. This can only be accurately described by the square displacement $r^2(t)$ of the particle, computed from its variance in both directions. Given that $d\left[x^2(t)\right]/dx = 2\,x(t)$ and $d\left[y^2(t)\right]/dy = 2\,y(t)$,

$$x^2(t) = 2V_d \int_0^t dt' \left[V_d \int_0^{t'} dt_1 + \int_0^{t'} dW_x(t_1)\right] + 2\int_0^t dW_x(t') \left[V_d \int_0^{t'} dt_1 + \int_0^{t'} dW_x(t_1)\right],$$

$$y^2(t) = 2\int_0^t dW_y(t') \left[\int_0^{t'} dW_y(t_1)\right].$$

When computing the expected value of these expressions for a time $t \gg dt$, the crossed terms cancel out (because the increments of the Wiener process are independent if evaluated over different intervals

of time). Hence, $\int_0^t dW_x \int_0^{t'} dW_x' = \int_0^t dW_x^2$. We thus get

$$\overline{x^2(t)} = V_d^2 t^2 + 2Dt, \qquad \overline{y^2(t)} = 2Dt.$$

From this follows the expression of the square displacement of the particle over time:

$$r^2(t) = V_d^2 t^2 + 4Dt. \tag{3}$$

Markov chain model

We focus on a coarse-grained description of the problem. Rather than following the individual trajectories of all mice, we concentrate on the occupation of each nest box. This has the advantage of reducing the complexity of the approach by not having to describe all individual movement equations. Moreover, the number of simulation variables thus stays constant (it depends solely on the number of nest boxes), whatever the number of agents in the simulation is. In other terms, we simulate the behaviour of the agents (mice) but observe only the result of their collective behaviour on the occupation density per box in the barn.

We describe the transitions from state to state (or here, from nest box to nest box) by a Markov chain (see Figure S1). The Markov assumption can be expressed at the agent level as follows: the conditional probability of an agent's future state depends only on its current state. In other words, the mice keep no memory of the boxes they visited prior to the one they currently stay in. The transition rate of a Markov process in the chain is the probability for an agent of leaving its nest box multiplied by the transit frequency from this nest box to any nest box, i.e. the joint probability of a mouse leaving its nest box for any nest box, including a return to the same nest box.

When going from a box to another, an agent needs a finite time. Moreover, we observe that for any 3 nest boxes i, j, k, the transit times from i to j and from i to k are different (according to a χ^2 test, normality hypothesis on the distribution of transit times rejected at a 0.05 confidence level for 23 boxes out of 40). To cover this specificity of our system, we need to introduce the concept of transit box (see Figure S1): whenever an agent is not in a nest box, we define that it is in an intermediary (transit) state between its origin and destination nest boxes, whose leaving rate depends on the particular origin and destination boxes. From here onwards, we regroup the terms "nest box" and "transit box" under the common denomination "box".

The N agents (the mice) are assumed to move within the discrete state-space formed by the B nest boxes and B^2 transit boxes according to stochastic processes. Therefore, each agent is uniquely described by its current box b. All processes are assumed to have the Markov property, with λ, Θ and Π being fixed parameters of the system.

- λ_i, $i \leq B$ is the leaving rate, a vector of length B. This is the rate at which an agent leaves nest box i aiming for another nest box, consequently entering the corresponding transit box. It is constructed from the data as the inverse of the mean leaving time per box.

- Θ is the transition rate, a square matrix of order $B + B^2$. The element $\Theta_{i,j}$ is the rate at which an agent leaves a transit box connected to node i to enter in the corresponding destination nest box j. It is constructed as the inverse of the mean transit time from any box to any other box, including itself.

- Π is the transition frequency, a square matrix of order B. This defines which transition actually occurs when an agent is leaving a nest box for another one, and accounts for the fact that transition probabilities between boxes are not equal. Π is row-stochastic, i.e. its row sum is always 1.

Master equation

In the mean field approximation, this system of $B + B^2$ elements can be considered as a one-dimensional occupation density vector and thus studied analytically by means of a system of master equations. For each state k, the probability P_k of being in such a state at a time t depends on the transition rates to and from this state and the occupation density of all the other states. In other words the variation of the density of agents in a particular state is the density of agents coming from other states from which the density of agents leaving for other states is subtracted:

$$\frac{\partial P_k}{\partial t} = \sum_{b=1}^{B+B^2} (T_{bk}P_b - T_{kb}P_k), \qquad \forall k \in \{1, \ldots, B + B^2\}, \tag{4}$$

where T is a $(B + B^2)$-square matrix of transition rates from any state to any other state, and T_{bk} is the transition rate from state b to state k. In our case, T is a composite of λ, Θ and Π, so that:

$$T_{ij} = \begin{cases} \Pi_{i,j-B\cdot i} \cdot \lambda_i, & j \in \{B \cdot i + 1, \ldots, B \cdot (i + 1)\} \\ \Theta_{i,j}, & i = B \cdot n + j \\ 0, & \text{otherwise} \end{cases}$$

where $\{i, j\} \in \{1, \ldots, B + B^2\}^2$, and $\forall n \in \{1, ..., B\}$. As there is only a restricted set of states reachable from any state (in fact, exactly B from the states corresponding to nest boxes and exactly 1 from those corresponding to transit boxes), T is mostly sparse.

This system of master equations and its asymptotic solution can be approached analytically, as is developed in the next section. Indeed, we find a good correspondence between the stationary state of our model and the observed data, which confirms that the Markov assumption holds in our problem. However, this analytical approach solely describes the "diffusion" of occupation densities, without characterising the actual encounters that happen inside the nest boxes visited. Beyond this diffusion process we want to characterise the outcome of our model on the social structure of the mice population. To this effect, we implement a full agent-based simulation of the Markov chain, seen from the perspective of the mice, as described in the main text.

Analytical approach to the stationary distribution

The master equation above describes the time evolution of occupation densities in the Markov chain model of our system. We want to compute the system's stationary state in order to test whether the Markov assumption holds. The transition matrix T does not formally describe a Markov chain, since it is not row-stochastic. We have to construct a row-stochastic transition matrix from T, in order to set a fixed timescale for our system: at each time step, a transition occurs. For all i and $j \in \mathbb{N}^2$, $T_{i,j}$ is the joint probability for an agent of leaving box i and entering box j thereafter; $\forall i$, $\sum_i T_{i,j}$ is the joint probability of leaving box i and entering any box, that is simply the probability of leaving box i. We need to include in the matrix T the possibility that an agent does not leave a box after one time step. If no leaving event has a rate (any of T row-sums) higher than $1\,\mathrm{s}^{-1}$, there is a straightforward manner to convert the known transition rates to transition probabilities. We set our time step to $1\,\mathrm{s}$ and copy all elements of T into a new matrix S. Since the rows of S do not sum up to 1, we define the probability for an agent of staying in the same state as the probability that it does not change to any other state, i.e. we obtain the stochastic matrix S as follows:

$$S_{ij} = \begin{cases} T_{ij}, & i \neq j \\ 1 - \sum_k T_{ik}, & i = k\ . \end{cases} \tag{5}$$

Because the mice stay rather long in a box, the leaving rate from each box is well below one, so the above mentioned condition applies. In the case where some events have a rate higher than $1\ \mathrm{s}^{-1}$, we would

have to normalise all rates to the highest and follow the same process. In the end, if i is the box with the fastest leaving rate, $S_{i,i} = 0$: the system's timescale has been adjusted to the fastest-happening event. S describes the complete Markov chain as illustrated by the dashed transition probabilities of Figure S1. The probability of moving from any state i to any state j during one time step is $Pr(j|i) = S_{i,j}$. Let d be the row vector of occupation density in all $B + B^2$ states. For any time t, we can write: $d_{t+1} = d_t \times S$, where d_{t+1} is the occupation density vector after one arbitrary time step. By recurrence, we can generalise this expression to the occupation density after t time steps:

$$d_t = d_0 \times S^t \ . \tag{6}$$

We want to find the stationary probability vector δ, i.e. the occupation density vector that does not change under application of the transition matrix. As stated by the Perron-Frobenius theorem [b], as our matrix is not strictly positive, there are several corresponding vectors (the eigenvectors corresponding to the dominant eigenvalue). We can however obtain a suitable δ as the limit of the application of S many times on any non-zero initial distribution. Let $d_0 \in \mathbb{R}^{B+B^2}$ characterise the initial distribution where all the occupation density is concentrated in box 1 and then slowly diffuses into all other boxes, $d_0 = [1, 0, \ldots, 0]$. We can numerically calculate δ:

$$\delta = \lim_{k\to\infty} d_0 \times S^k \ , \tag{7}$$

Each of the first B coefficients of δ is the stationary occupation density of a nest box, normalised to 1. To relate it to our observed occupation times, we would have to multiply each coefficient of the vector by the number of time steps the simulation covers and the mean number of agents in the system.

Numerical computations confirm that the simulated occupation density vector δ converges towards the experimental occupation density measured δ^* with a very high precision (Pearson's correlation test $P < 10^{-10}$ after a certain number of iterations, always less than our time range of two years), regardless of the initial distribution. This is illustrated in Figure S2 and Table S1 in the case where the initial occupation is concentrated in box 1.

Supplementary references

a. Murray JD (2002) Mathematical Biology: I. An Introduction. Springer.

b. Seneta E (2006) Non-negative Matrices and Markov Chains (Springer Series in Statistics). Springer.
